# Hepatitis – a devastating epidemic in Europe

**DOI:** 10.1186/1471-2334-14-S6-S1

**Published:** 2014-09-19

**Authors:** Jeffrey V Lazarus, Kevin A Fenton

**Affiliations:** 1 CHIP, Centre for Health and Infectious Disease Research and WHO Collaborating Centre on HIV and Viral Hepatitis, Rigshospitalet, University of Copenhagen, Copenhagen, Denmark; 2 Public Health England, London, United Kingdom

## 

With hepatitis B and C, the European region is confronting a devastating epidemic that seemingly has emerged in silence. The major driver of hepatitis B and C in Europe is now injecting drug use. Yet it would be a mistake to think of these diseases as a problem primarily for people who inject drugs (PWID). They are a problem for governments, health systems, communities, families and all who care about the rights and dignity of the most marginalised members of our society.

The first European Conference on Hepatitis C and Drug Use, slated for 23-24 October 2014, may well be seen in retrospect as a landmark event spurring the emergence of the broad-based movement that is needed to alter the course of the region’s hepatitis epidemic. The conference’s international organising partner, the Correlation Network’s Hepatitis C Initiative, has utilised financial support from the Drug Prevention and Information Programme of the European Union to publish a journal supplement in conjunction with the conference. We, the editors of the supplement, are proud to have had this opportunity to bring together information and viewpoints about some of the most important issues on the conference agenda. We trust that the publication will both inform the current dialogue around key issues and also find a place as a core resource for the many additional people who will surely be drawn to this work in the years to come.

The European Conference on Hepatitis C and Drug Use takes place at a pivotal time. The first-ever World Health Assembly resolution on viral hepatitis in 2010 [1] generated momentum in relation to key strategy and policy issues at the global level, as reflected in the viral hepatitis strategic framework announced by the World Health Organization (WHO) in 2012 [2]. In 2013, WHO followed up by asking Member States to report on existing national policies and practices in relation to the four axes of the strategic framework. The resulting *Global Policy Report on the Prevention and Control of Viral Hepatitis in WHO Member States *[3] provides a benchmark for measuring progress on a number of critical issues.

In the spring of 2014, WHO’s hepatitis programme convened its first-ever “global partners meeting,” and the resulting “Call to Action to Scale Up Global Hepatitis Response” captured the major concerns of the many civil society organisations that attended the meeting [4]. Not long afterwards, strong civil society engagement helped to ensure passage of a new World Health Assembly resolution [5] that obligates governments to take more concrete measures in response to viral hepatitis. Civil society again came together to contribute to the first *Global Community Hepatitis Policy Report*, released by the World Hepatitis Alliance on World Hepatitis Day 2014 [6].

The importance of addressing the viral hepatitis prevention and treatment needs of people who inject drugs has been a strand running through all of these developments. Indeed, one of the key advocacy victories associated with the new World Health Assembly resolution is the inclusion of commitments relating to people who inject drugs. The resolution calls on WHO Member States to “ implement comprehensive hepatitis prevention, diagnosis and treatment programmes for people who inject drugs.” Footnoted text names nine key interventions, including needle and syringe programmes; opioid substitution therapy and other drug dependence treatment; and targeted information, education and communication for people who inject drugs and their sexual partners.

In short, this is an opportune time to bring PWID issues to the forefront in discussions about how to rein in hepatitis B and C in Europe and globally.

The discussions underway in policy, advocacy and research circles are by necessity very broadly focused. Viral hepatitis prevention efforts must overcome immense obstacles associated with low public awareness and misconceptions about the nature of the threat. The same factors hinder health systems from identifying a vast number of infected but currently asymptomatic and undiagnosed people who might benefit from treatment. There is also much to be resolved around fundamental matters such as uniform disease surveillance standards and treatment initiation criteria. The hepatitis C treatment cascade needs to be more clearly conceptualised so that vital questions can be pursued regarding how to engage, retain and successfully treat far more people.

PWID issues are not an “add-on” to this already daunting agenda. They are cross-cutting throughout the agenda, for the simple reason that people who inject drugs are commonly the human face of the hepatitis B and C epidemics in many countries, including a large number of countries in Europe. Successfully engaging these individuals in prevention and treatment efforts means addressing a complex public health threat within the context of other equally complex threats – such as addiction, mental illness, financial instability and the stigmatisation and criminalisation of drug users.

The WHO European region has an estimated 15 million people living with hepatitis C, of whom an estimated two million are current drug injectors [7]. At the same time, only 30% of European countries submitting information to WHO for the 2013 policy report indicated that they have comprehensive national viral hepatitis strategies or plans. Seventy percent told WHO that they have hepatitis prevention policies targeting PWID. With such large gaps in national leadership, it is no wonder that the overall public health response to the viral hepatitis epidemic among PWID appears to be so fragmented.

Yet the lack of a high-profile discourse regarding how to elaborate the strategies suggested by the WHO framework should not be interpreted as a lack of interest in hepatitis and PWID. As the contributions to this supplement suggest, a vast array of people and organisations have been quietly forging ahead in their own communities and peer networks. The collective wisdom accruing through these efforts must be shared more widely, and it must be shared as quickly as possible – there is absolutely no time to lose.

In this context, the supplement editors hope that the publication and conference together help usher in a new era of knowledge-sharing among those who are committed to preventing and treating hepatitis B and C among people who inject drugs. We are pleased to note that the supplement presents no less than ten commentaries about crucial issues in this realm, and we are equally pleased about the diversity of experiences represented in the contributions. To illustrate:

• In “No Strategy to Meet the HCV Epidemic,” Olav Dalgard and Stefan Mauss of the European Association for the Study of the Liver discuss some of the vast implications of the changing hepatitis C treatment paradigm, including the opportunity to raise awareness about prevention.

• Achim Kautz and colleagues at the European Liver Patients Association broadly outline key strategic considerations in relation to improving hepatitis C screening and treatment for PWID in Europe.

• Chris Ford and Juliet Bressan, United Kingdom-based physicians with a wealth of knowledge about addiction services, argue forcefully for the decriminalisation of people who use drugs as a strategic element of the hepatitis C response.

• In “A Treatment Revolution for Those Who Can Afford It?” Maria Phelan and Catherine Cook of Harm Reduction International capture some of the central issues being raised by the extremely high price of new direct-acting antiviral drugs and call attention to what this means for PWID in Europe.

• Slovenia’s exemplary national multidisciplinary healthcare network for treating hepatitis C in people who inject drugs is described by physician Mojca Maticic, a hepatitis expert who has played a major role in helping to develop the Slovenian model.

• Leon Wylie of Hepatitis Scotland and other key actors involved in the Scottish Hepatitis C Action Plan identify ways in which the acclaimed Scottish approach can serve as a valuable example for others seeking to develop effective national and pan-European responses to hepatitis C.

• Astrid Leicht of Fixpunkt, a German nongovernmental organisation working to promote the health of people who use drugs, demonstrates the expertise that those working at the community level are ready to contribute in “Improving the Quality of Needle and Syringe Programmes: an Overlooked Strategy for Preventing Hepatitis C among People Who Inject Drugs.”

• Ricardo Baptista Leite, a physician and Member of Parliament in Portugal, explains the process that led to the strategic consensus for the integrated management of hepatitis C in Portugal and highlights primary recommendations.

• Two contributions from Spain both look critically at *ECDC and EMCDDA Guidance: Prevention and Control of Infectious Diseases among People Who Inject Drugs *[8]. Antonio Corbacho and co-authors representing five Spanish drug user organisations share insights about what this landmark guidance document means for their constituencies, as well as identifying shortcomings and proposing addenda that reflect drug users’ concerns. In a similar vein but from a quite different vantage point, Joan Colom i Farran of the Public Health Agency of Catalonia offers a policy-maker’s perspective on *ECDC and EMCDDA Guidance*, and in doing so draws out elements of the guidance that warrant more careful consideration.

The publication also contains five research articles and a discussion article that collectively demonstrate the potential for smart research to help transform the political and public health response to hepatitis C in Europe. Robert Heimer and colleagues present findings from a bio-behavioural study on hepatitis C among PWID in eight Russian cities, calling attention to the advanced epidemic there. In “HIV and hepatitis C Co-infection in Europe, Israel and Argentina: a EuroSIDA Perspective,” Lars Peters and colleagues examine the nested hepatitis cohort in one of the world’s major HIV cohort studies. Mojca Maticic and colleagues present findings that will be valuable to advocates and policy-makers in their article, “Are There National Strategies, Plans and Guidelines for the Treatment of Hepatitis C in People who Inject Drugs? A Survey of 33 European Countries.”

Alexander Spina and colleagues focus on the PWID findings from the aforementioned first WHO global hepatitis policy report in “Policy Responses to Viral Hepatitis B and C among People Who Inject Drugs in Member States of the WHO European Region: a Sub-analysis of the WHO 2013 Global Hepatitis Policy Survey.” Jeffrey V Lazarus and colleagues report worrisome findings about low treatment levels in “A Systematic Review of Hepatitis C Virus Treatment Uptake among People Who Inject Drugs in the European Region,” while Amber Arain and colleagues provide recommendations regarding hepatitis C prevention, screening and treatment in prisons, a highly neglected setting.

Finally, the supplement presents a timely roundtable discussion entitled “How Lessons Learned from HIV can Inform the Global Response to Viral Hepatitis”" with patient, physician, epidemiologist, qualitative researcher and grassroots advocate perspectives.

What the editors find most striking about this unique collection of pieces is that it embodies an incredible wealth of knowledge drawn from important front-line work. When we say this, we are referring to *all* of the front lines that are so important in addressing the hepatitis C epidemic in PWID communities. Many contributors to this supplement are speaking on the basis of their own direct experience grappling with enormous challenges – as people who use or formerly used drugs, as people who provide health care, addiction treatment, social support and peer support to drug users, as researchers looking for innovative ways to acquire evidence about an often-inaccessible population, and as activists, advocates and policy-makers who are fighting to wake up others regarding the threat posed by hepatitis C and the moral imperative to respond without judging anyone who is at risk.

Furthermore, many contributors transcend what might be thought of as their proscribed roles as activists, researchers, physicians, policy-makers and so on. We hope that this approach is reflective of what is happening in the overall European response to hepatitis and PWID, because it is exactly what is needed: physicians must engage in policy issues, activists must persuade others to support the most appropriate strategies and interventions by using the dispassionate logic that is characteristic of researchers, and public health administrators must inspire change by displaying the passion of activists.

Pragmatism emerges as an important theme of the supplement. For example, as much as good evidence is required to inform the response to hepatitis C in Europe, Joan Colom i Farran reminds readers of why it can also be pragmatic to move forward on merely the basis of good common sense. The author calls for supervised injecting facilities to be emphasised much more in any future edition of the *ECDC and EMCDDA Guidance*, noting:

*I am aware that research on those facilities is limited*, *but do you really need much research to prove that injecting inside these facilities is much safer than injecting elsewhere? It is like needing to prove that jumping from a plane with a parachute is safer than jumping without one*, *is it not?*

As this observation reminds us, a key to navigating so many strategic considerations relating to hepatitis prevention and treatment for PWID is knowing when more evidence is needed and when it is time to act on the limited available evidence in the interest of trying to save as many lives as possible. As all types of stakeholders grapple with this issue in the years to come, we hope that cues will be taken from the invaluable insights of the most at-risk and most affected communities. In that spirit, we would like to conclude by quoting from the commentary provided by the Spanish drug user organisations:

*It would be difficult to find another at-risk population whose needs have been so widely ignored…. Give us voice and respect*, *more often and in more situations*, *and we are sure that this will make a difference in the prevention of drug-related harm*, *including harm from the transmission of hepatitis C and other bloodborne viruses.*

## Competing interests

The authors declare that they have no conflicts of interest.

## Declarations

This article has been published as part of *BMC Infectious Diseases* Volume 14 Supplement 6, 2014: Viral Hepatitis in Europe. The full contents of the supplement are available online at http://www.biomedcentral.com/bmcinfectdis/supplements/14/S6. The publication charges for this supplement were funded by AbbVie as an unrestricted grant to Rigshospitalet, the University of Copenhagen. AbbVie further funded the printing of the supplement with additional financial support from the Drug Prevention and Information Programme (DPIP) of the European Union.
